# Obituary: Dr Dimitri Tassiopoulos

**DOI:** 10.1080/17290376.2017.1334288

**Published:** 2017-06-22

**Authors:** Crain Soudien

**Affiliations:** ^a^ Human Sciences Research Council


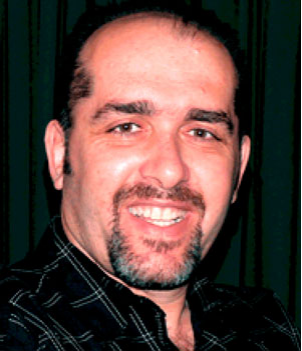


It is with my deepest sorrow that I announce the untimely death of the passing of Dr Dimitri Tassiopoulos.

Dr Dimitri Tassiopoulos was a Chief Research Manager with Social Aspects of HIV/AIDS Research Alliance (SAHARA) within the HIV/AIDS, STIs and TB (HAST) research programme of the HSRC. He held a BA (Hons.) degree in Political Science from the University of Stellenbosch, and obtained an MBA and PhD in Management and Administration from the University of Stellenbosch Business School in South Africa.

Before joining the HSRC in May 2011, he was an Associate Director at the School of Tourism and Hospitality at the Walter Sisulu University (WSU) in East London, South Africa. He spent the majority of his academic career as a head of a School or as a Departmental Chairperson. After 1993, he was involved in various national and international tourism research projects, of a multidisciplinary and multi-institutional nature, concerning agritourism, event, cultural and wine tourism, amongst others. In 2004 he was appointed as a member of the South African Qualifications Authority’s Standards Generating Body (SGB) for Travel, Tourism and Events, and was appointed as its chairperson. He was the vice president of the Tourism and Hospitality Education Providers of South Africa (THEPSA). This Federation represents further (FET) and higher education (HET) institutions offering the spectrum of qualifications related with the management of the tourism industry. He was also the chairperson of the Tourism and Events Educators Chamber of South Africa (TEECSA), an association representing higher education travel, tourism and events related programme providers.

Dr Tassiopoulos successfully supervised post-graduate mini-theses/theses/dissertations and served as an external examiner for post-graduate theses at various universities in South Africa. Dr Tassiopoulos was a board member of a number international peer-reviewed research journals. The evidence of Dr Tassiopoulos’s work is reflected in his publication record that spans the authoring and co-authoring of more than 16 presentations, at local, regional and international conferences, and, nine scientific publications in peer-reviewed journals. He was awarded six grants for research, published six books, published 33 chapters in books, has been involved in seven commissioned research projects. He has been rated a C3 researcher by the National Research Foundation. He served as a book reviewer for a number of publishers and as a proposal reviewer for the National Research Foundation.

Dr Tassiopoulos was involved as an International Reviewer for the European Council on Hotel, Restaurant and Institutional Education (EuroCHRIE), Dubai 2008 Conference. He was involved as a session chair for the 2008 International Council for Small Business (ICSB) Conference, Halifax, NS, Canada; and, the 7th International Conference for Consumer Behaviour and Retailing Research (CIRCLE), Estoril Higher Institute for Tourism and Hotel Studies (ESHTE), Estoril, Portugal. He served on the following journal editorial boards as a board member of a number international peer-reviewed research journals. In particular, *Tourism Today*: published annually by the College of Tourism and Hotel Management, *Nicosia, Cyprus* (ISSN 1450-0906) (http://www.cothm.ac.cy/research.htm), and *European Journal of Tourism Research*: an academic refereed journal published bi-annually by International University College, Bulgaria (ISSN: 1994-76580) (http://ejtr.vumk.eu/). He was a regular reviewer of articles submitted to the prestigious *Tourism Management Journal* for publication.

Among some of his achievements since joining HSRC Dr Tassiopoulos served as Managing Editor of *SAHARA-J: Journal of Social Aspects of HIV/AIDS* (SAHARAJ). He successfully, while working closely together with Prof. Phaswana-Mafuya, managed to bring the journal under the Taylor & Francis Online stable with Routledge Press. SAHARAJ’s impact factor has risen during his leadership. Dr Tassiopoulos also managed the SAHARA Conference.

Dr Tassiopoulos was involved in collaborating in cross-disciplinary research projects. He was involved in researching the effects of HIV and AIDS within higher education and the management of this syndrome that is afflicting the higher education population. More recently, he was involved in co-editing and publishing a book entitled non-communicable diseases in developing countries. Most recently, he led both international and South African research in the field of HIV and the World of Work Initiatives.

Dr Dimitri Tassiopoulos made a significant contribution to South Africa’s social science capital and his bright mind, dedication and passion will be missed. I take this opportunity again to convey my condolences to all of you who have known and worked with Dr Dimitri Tassiopoulos.

